# Accounting for focal shift in the Shack–Hartmann wavefront sensor

**DOI:** 10.1364/OL.44.004151

**Published:** 2019-09-01

**Authors:** Vyas Akondi, Alfredo Dubra

**Affiliations:** Byers Eye Institute, Stanford University, Palo Alto, California 94303, USA

## Abstract

The Shack–Hartmann wavefront sensor samples a beam of light using an array of lenslets, each of which creates an image onto a pixelated sensor. These images translate from their nominal position by a distance proportional to the average wavefront slope over the corresponding lenslet. This principle fails in partially and/or non-uniformly illuminated lenslets when the lenslet array is focused to maximize peak intensity, leading to image centroid bias. Here, we show that this bias is due to the low Fresnel number of the lenslets, which shifts the diffraction focus away from the geometrical focus. We then demonstrate how the geometrical focus can be empirically found by minimizing the bias in partially illuminated lenslets.

The Shack–Hartmann wavefront sensor (SHWS) is used to measure the departure of test wavefronts from a typically flat or spherical reference wavefront [1]. Originally conceived for metrology [2], the SHWS has now been adopted in multiple fields including astronomy [3], line-of-sight communications [4], microscopy [5], high-power laser systems [6], retinal imaging [7–9], and refractive surgery [10].

In essence, a SHWS consists of a pixelated light detector in the back focal plane of a two-dimensional array of lenslets [1]. The light reaching the lenslets creates an array of images onto the pixelated sensor. According to geometrical optics, each individual image will translate by a distance proportional to the wavefront slope over the corresponding lenslet (Fig. 1). Fresnel diffraction theory, however, provides more insight into the relation between the distance *z* of the pixelated sensor from the geometrical focus, the lenslet image centroid vector position ***ρ***_*c*_ (*z*), the light intensity at the lenslet plane *I* (***r***), the wavefront gradient ∇*W***(*r*)**, and the geometrical focal length, *f*, of the lenslet [11]:

(1) $$\rho_{c}\left(z\right)=-\frac{z}{f}\rho_{c}\left(-f\right)+\left(f+z\right)\frac{\int I\left(r\right)\nabla W\left(r\right){\text{d}}^{2}r}{\int I\left(r\right){\text{d}}^{2}r}.$$

In this equation, ***ρ***_*c*_(−*f*) is the centroid of the light intensity at the lenslet plane, *z* = −*f*, and ***r*** is the transverse position vector. The first term on the right-hand side of the equation does not contain information about the wavefront, and thus can be thought of as an undesired bias term.

Most SHWSs use lenslets that are symmetric relative to their centers (e.g., hexagonal or square) and are uniformly illuminated (i.e., *I* **(*r*)** *I*_0_). For these lenslets, the bias term vanishes, as ***ρ***_***c***_(−*f*) = 0 if we assume that the lenslet center is at the coordinate system origin, and thus Eq. (1) reduces to the widely used relation

(2) $$\rho_{c}\left(z\right)=\left(f+z\right)\frac{\int \nabla W\left(r\right){\text{d}}^{2}r}{\int {\text{d}}^{2}r}.$$

This is effectively, the SHWS principle, which is valid even if the sensor is not in the focal plane of the lenslet array, provided the lenslets are center-symmetric and uniformly illuminated.

The SHWS principle, however, may fail in two ways in non-uniformly illuminated lenslets: first, by unevenly weighting the gradient across the lenslet, through the wavefront term in Eq. (1), unless the wavefront gradient can be considered constant, in which case, this term is immune to the illumination profile. Second, and more importantly, is the case in which the pixelated sensor is not at the geometrical focus of a lenslet (i.e., *z* ≠ 0), and is unevenly illuminated (i.e., ***ρ***_*c*_(−*f*) ≠ 0), thus producing a non-zero bias term, as can be seen from Eq. (1). This bias is most evident at the boundary of the test beam, where lenslets are partially illuminated.

A brief survey of scientific publications [7,12–36] and commercial instruments reveals SHWS lenslet arrays with a Fresnel number (*N = a*^2^∕*λf*, with *a* the aperture radius and *λ* the wavelength of light) in the 0.47–9.3 range. In this low Fresnel number regime, the point of maximal intensity behind the lenslet (the diffraction focus) is shifted toward the lenslet and away from the geometrical focus [2,37–39]. This relative focal shift can be approximated as

(3) $$\frac{\Delta f}{f}≊-\frac{12\left(1+\frac{1}{8F^{2}}\right)}{\pi^{2}N^{2}}\times \left\{1-\text{exp}\left[-\frac{\pi^{2}N^{2}}{\left[12\left(1+\frac{1}{8F^{2}}\right)\right]}\frac{1}{\left[1+N\left(1-\frac{1}{16F^{2}}\right)\right]}\right]\right\},$$

where *F = f* ∕2*a* ≥ 0.5 and the Fresnel number *N >* 1 [38,39]. Assuming 850 nm light, the range of relative focal shifts in the surveyed lenslet arrays is as small as 1% and more than 50%.

The resulting centroid bias in partially illuminated lenslets could, in principle, be corrected through a careful calibration in which the beam used for defining the reference wavefront had the same intensity profile as the test beam, and in particular, at the partially illuminated lenslets. Unfortunately, this is not practical when the calibration beam covers a larger area of the lenslet array than the test beam. In ophthalmic SHWSs, such calibration is not even possible, as the pupil of the eye is in constant motion. A simpler and arguably more natural method for mitigating the centroid bias would be to focus the pixelated sensor in the geometric focal plane of the lenslet array, as we demonstrate next.

An experimental setup depicted in Fig. 2 was used to create a collimated beam by placing the tip of a single-mode optical fiber a focal length away from an achromatic doublet. A lenslet array-conjugated adjustable iris diaphragm was fully open to overfill the lenslet array such that all the lenslets were fully illuminated, i.e., when ***ρ***_***c***_(−*f*) = 0, and therefore, the first term on the right-hand-side of Eq. (1) was zero. This allowed us to iteratively collimate the beam by moving the fiber axially until the SHWS spot displacement in response to translating the pixelated sensor axially was minimized. This collimation made the wavefront-dependent term in Eq. (1) effectively zero, leaving only a bias term.

The adjustable iris was then closed to create partially illuminated lenslets that exhibited a centroid shift proportional to the distance of the pixelated sensor from the lenslet geometrical focus. In this way, the SHWS pixelated sensor was axially translated until the spot displacement of the partially illuminated lenslets was minimized.

Visualization 1 (see Fig. 3) shows how the SHWS spots shift as the iris is closed with the pixelated sensor in the diffraction and geometrical foci side by side. As the iris closes, the outermost spots correspond to partially illuminated lenslets. In the diffraction focus, the centroid of the spots corresponding to partially illuminated lenslets moves toward the center of the pupil, as predicted by the bias terms in Eq. (1), even though the wavefront itself does not change when the iris is closed. We found that the camera sensor had to be translated by 1.5 mm away from the position of maximal spot peak intensity, which is consistent with the 1.44 mm predicted by Li [38], 1.7 predicted by Sheppard and Török [39], and 1.36 mm from our numerical calculation based on Li’s diffraction integral [40]. For this lenslet array, 1.5 mm corresponds to a 23% relative focal shift.

The spot centroid displacements for the 60 frames in Visualization 1 are plotted as a function of peak spot intensity in Fig. 4. Only pixels with intensity higher than 5% and lenslets with peak intensity higher than 10% of the camera dynamic range were included in the plot and subsequent analyses. At the geometric focus, we find that for the standard deviation of all spot displacements in partially illuminated lenslets, defined as with total number of photons below 95% of when fully illuminated, the centroid bias is reduced on average by 63%, relative to the diffraction focus. Complete bias cancellation was not achieved, likely due to imperfect collimation, reaching the centroid repeatability limit in our setup and the lenslet crosstalk due to the diffraction-enlarged spots in partially illuminated lenslets. When considering the two clusters of points on the right side of Fig. 4 that correspond to fully illuminated lenslets, the mean centroid displacement for the diffraction focus is ~26% higher than for the geometric focus, but within the repeatability of our experimental setup. Therefore, it is not possible at this point to draw conclusions about the origin of this difference.

The sequence of SHWS images also shows that, as expected, the spots at the diffraction focus are narrower and brighter (see Fig. 5), while at the geometric focus, spots are broader and have broader minima that reach lower values. Fresnel diffraction theory indicates that at the geometric focus of a lenslet, the intensity minima are zero [37], but the crosstalk from diffraction patterns from adjacent lenslets and the finite pixel size can prevent this condition from being met.

Finally, and in order to illustrate the impact of the bias term, we integrated the centroid displacement maps from Visualization 1 as if they were due to real wavefront slopes, using the vector matrix multiply method and the slope geometry by Southwell [41]. The root-mean-square (RMS) of the calculated wavefront after removing the piston, tip, and tilt for all the frames in the video were calculated and are shown in Fig. 6. The video-averaged wavefront RMS when partially illuminated lenslets are considered is 0.06 ± 0.02 (mean ± std.dev.) waves at the diffraction focus, which is reduced to 0.02 ± 0.01 waves at the geometric focus. The wavefront RMS error at the diffraction focus is significant because it can be above Marechal’s criterion for a diffraction-limited optical instrument. Excluding the partially illuminated lenslets, the wavefront RMS dramatically reduces to 0.007 ± 0.003 waves and 0.004 ± 0.001waves at the diffraction and geometric foci, respectively. This indicates the vast majority of the artifactual wavefront RMS is a result of artifactual centroid bias that originates at the partially illuminated lenslets.

In summary, we have identified the focal shift due to diffraction as a reason for SHWS centroid bias in partially illuminated lenslets and have demonstrated its correction by shifting the pixelated detector to the geometrical focus of the SHWS lenslet array. The presence of this uncorrected bias in pupil edge lenslets has typically been addressed by ignoring these lenslets in wavefront estimation algorithms. This results in poor wavefront estimation at the pupil edges, with its impact varying across applications and wavefronts. In adaptive optics for imaging applications, achieving the best possible wavefront correction at the pupil edge is critical, as the photons at the pupil edge carry the highest spatial frequencies that determine the smallest resolvable objects.

## Figures and Tables

**Fig. 1.**
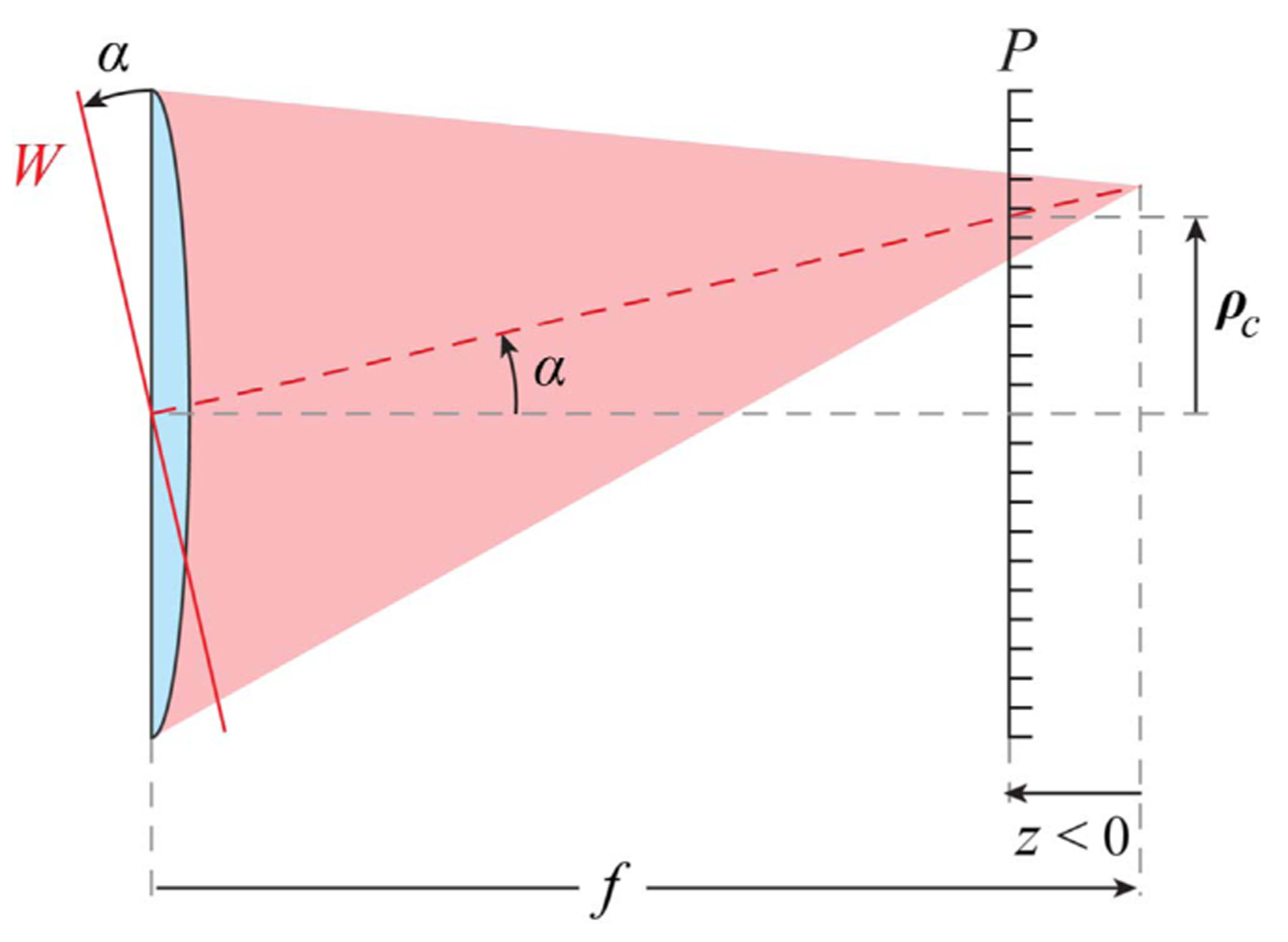
Geometrical SHWS principle: the transverse centroid (*x* and *y* components) position ***ρ***_***c***_(*z*) of the image formed by a lenslet of focal length *f* on a pixelated sensor *P* is proportional to the slope, *α*, of the incident wavefront *W*.

**Fig. 2.**
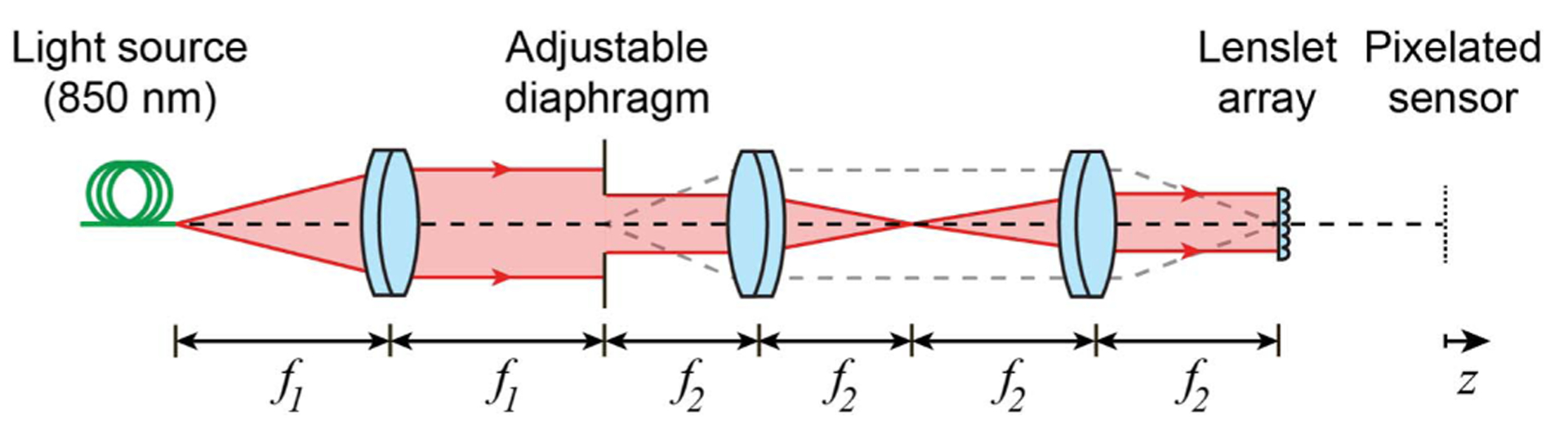
Experimental setup for source collimation and SHWS pixelated sensor focusing, in which *f*_1_ = 250 mm, *f*_2_ = 150 mm, *f*_lenslet_ = 6.5 mm, and lenslet width is 203 μm. Here, the gray dashed rays indicate optical conjugation between the iris and the lenslet array.

**Fig. 3.**
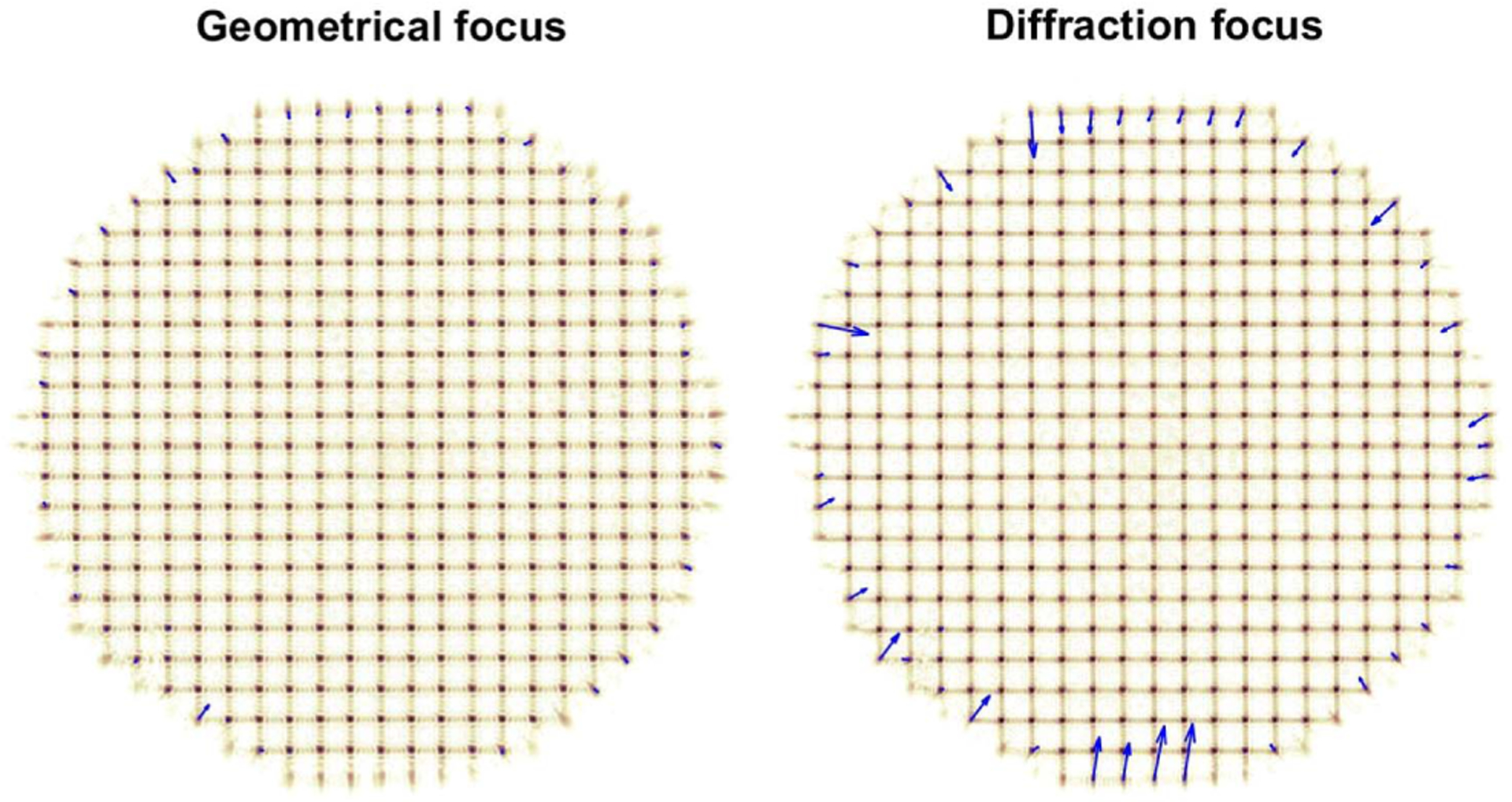
Sequence of SHWS images captured with the experimental setup in Fig. 2 while the iris is closing and with the pixelated detector at the geometrical focus (left) and diffraction focus (right) of the lenslet array (see Visualization 1). The blue arrows show the spot displacements from their nominal position, magnified 20 times for display purposes. The displacements of outermost spots, which correspond to partially illuminated lenslets, are substantially larger at the diffraction focus.

**Fig. 4.**
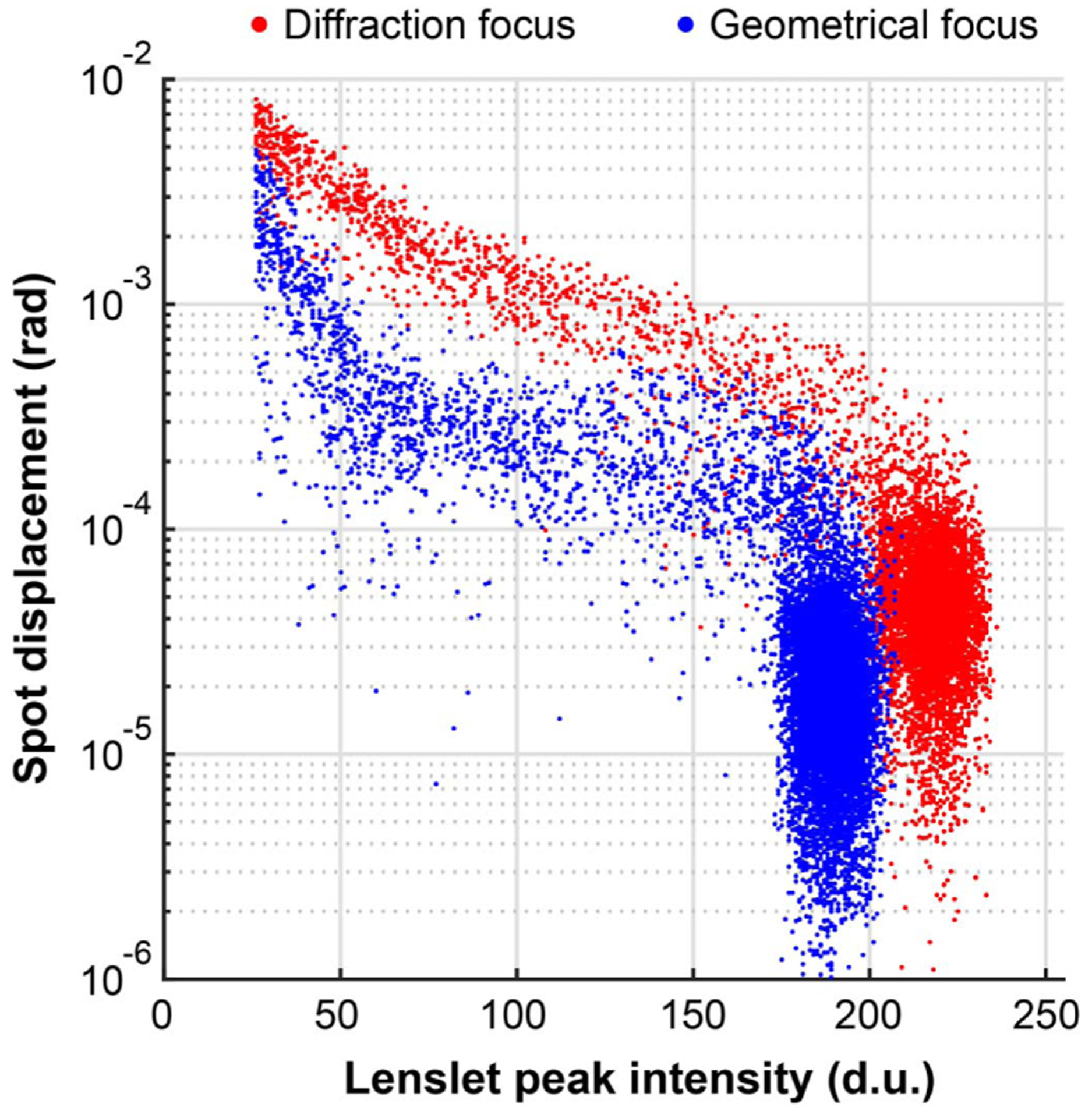
SHWS spot displacements from two image sequences captured using the experimental setup in Fig. 2 while the iris is being closed, as a function of spot peak intensity (see Visualization 1).

**Fig. 5.**
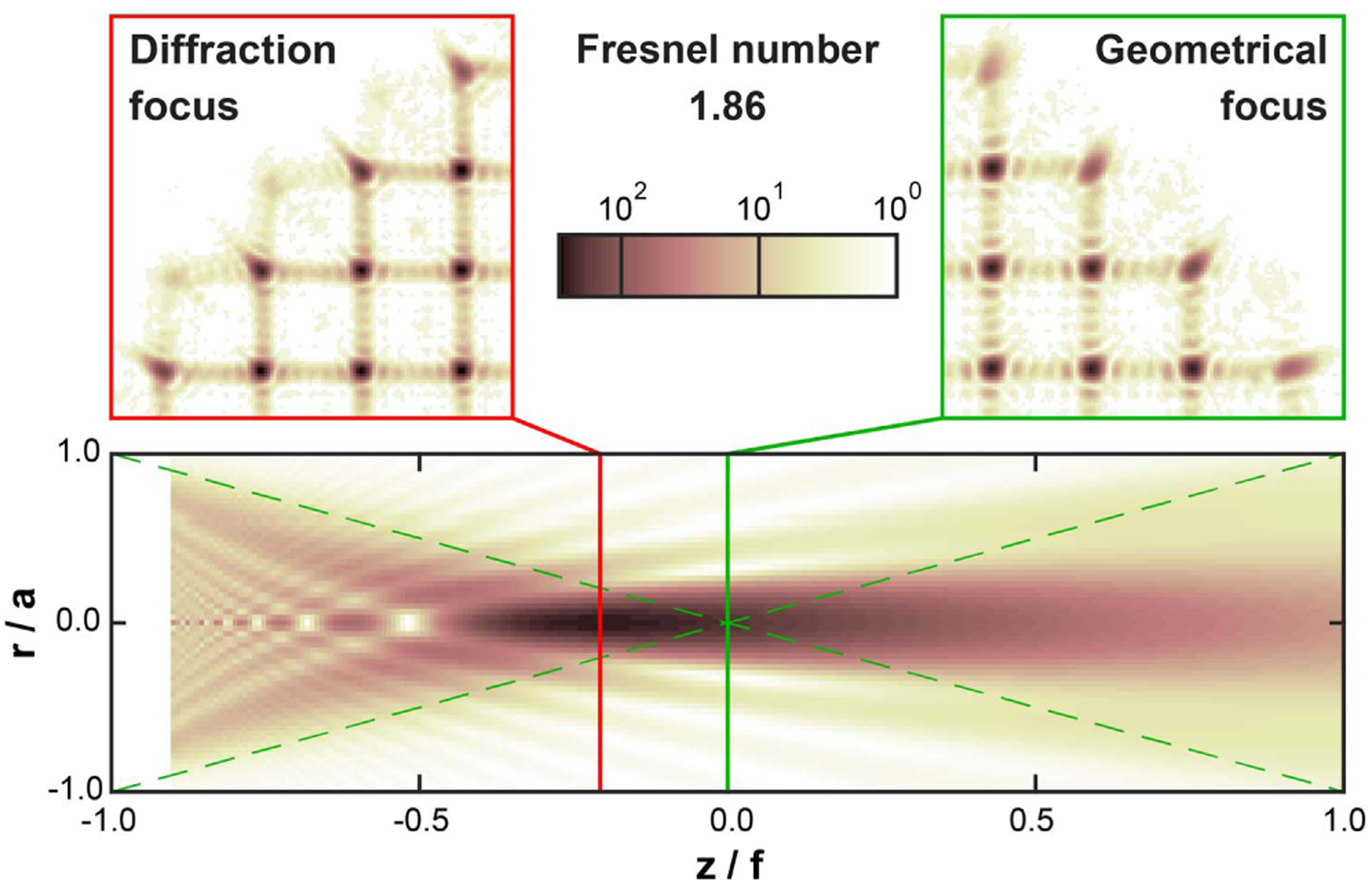
Magnified SHWS images with the pixelated detector at the lenslet array diffraction and geometrical foci, displayed in a logarithmic intensity scale. Note that the spots from partially illuminated lenslets exhibit not only lower peak intensity, but also a clear radial diffraction “tail.” The lower panel shows the calculated image intensity [40] on a meridional plane for a single uniformly illuminated lenslet with a circular aperture, in which the point of maximal intensity (diffraction focus) is shifted toward the lenslet (left) from the geometrical focus. The dashed lines in this panel show the geometrical cone for reference.

**Fig. 6.**
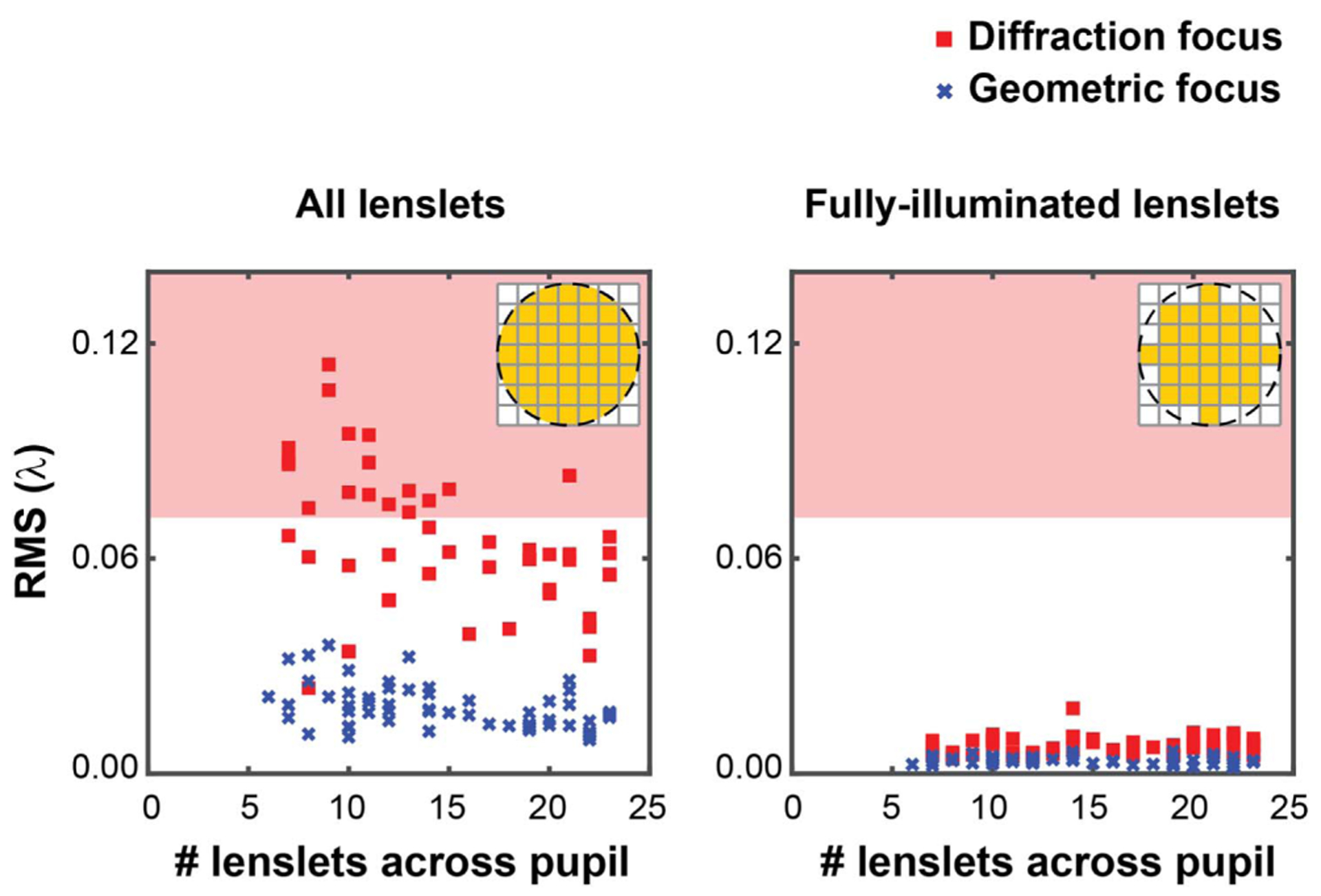
Wavefront RMS in waves for all 60 frames of the Visualization 1 video calculated including (left) and excluding (right) partially illuminated lenslets. The red shaded regions represent wavefront RMS greater than the diffraction limit (*λ*/14) of Marechal’s criterion.
